# Myasthenia Gravis: From the Viewpoint of Pathogenicity Focusing on Acetylcholine Receptor Clustering, Trans-Synaptic Homeostasis and Synaptic Stability

**DOI:** 10.3389/fnmol.2020.00086

**Published:** 2020-05-28

**Authors:** Masaharu Takamori

**Affiliations:** Neurological Center, Kanazawa-Nishi Hospital, Kanazawa, Japan

**Keywords:** neuromuscular junction, myasthenia gravis, acetylcholine receptor, agrin, wnts, MuSK, Lrp4, matrix proteins

## Abstract

Myasthenia gravis (MG) is a disease of the postsynaptic neuromuscular junction (NMJ) where nicotinic acetylcholine (ACh) receptors (AChRs) are targeted by autoantibodies. Search for other pathogenic antigens has detected the antibodies against muscle-specific tyrosine kinase (MuSK) and low-density lipoprotein-related protein 4 (Lrp4), both causing pre- and post-synaptic impairments. Agrin is also suspected as a fourth pathogen. In a complex NMJ organization centering on MuSK: (1) the Wnt non-canonical pathway through the Wnt-Lrp4-MuSK cysteine-rich domain (CRD)-Dishevelled (Dvl, scaffold protein) signaling acts to form AChR prepatterning with axonal guidance; (2) the neural agrin-Lrp4-MuSK (Ig1/2 domains) signaling acts to form rapsyn-anchored AChR clusters at the innervated stage of muscle; (3) adaptor protein Dok-7 acts on MuSK activation for AChR clustering from “inside” and also on cytoskeleton to stabilize AChR clusters by the downstream effector Sorbs1/2; (4) the trans-synaptic retrograde signaling contributes to the presynaptic organization *via*: (i) Wnt-MuSK CRD-Dvl-β catenin-Slit 2 pathway; (ii) Lrp4; and (iii) laminins. The presynaptic Ca^2+^ homeostasis conditioning ACh release is modified by autoreceptors such as M1-type muscarinic AChR and A2A adenosine receptors. The post-synaptic structure is stabilized by: (i) laminin-network including the muscle-derived agrin; (ii) the extracellular matrix proteins (including collagen Q/perlecan and biglycan which link to MuSK Ig1 domain and CRD); and (iii) the dystrophin-associated glycoprotein complex. The study on MuSK ectodomains (Ig1/2 domains and CRD) recognized by antibodies suggested that the MuSK antibodies were pathologically heterogeneous due to their binding to multiple functional domains. Focussing one of the matrix proteins, biglycan which functions in the manner similar to collagen Q, our antibody assay showed the negative result in MG patients. However, the synaptic stability may be impaired by antibodies against MuSK ectodomains because of the linkage of biglycan with MuSK Ig1 domain and CRD. The pathogenic diversity of MG is discussed based on NMJ signaling molecules.

## Introduction

**Figure 1:**
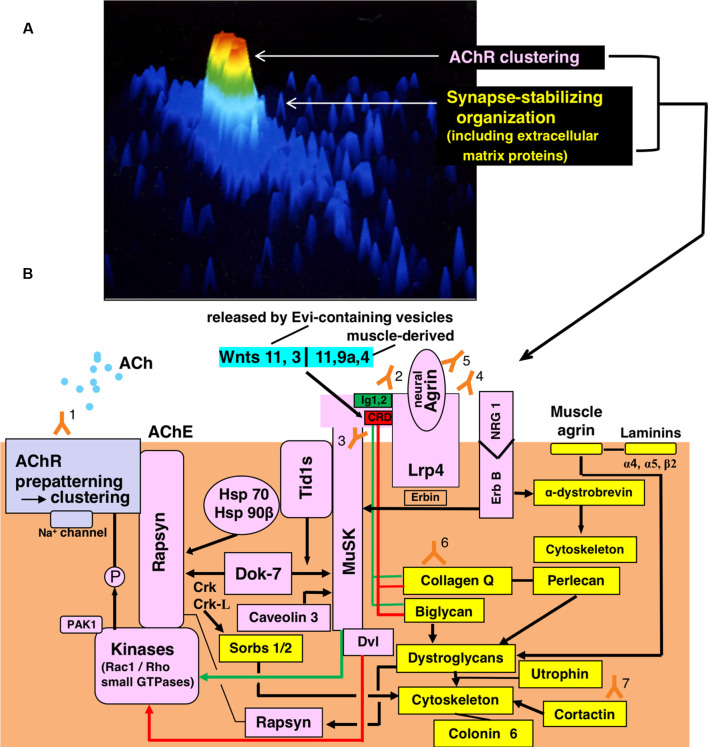
Functional organization for synaptic transmission in neuromuscular junction (NMJ) and antibody-targets. **(A)** Presentation by staining of cultured rat myotube with fluorescence-labeled α-bungarotoxin and by the image analyzing using a laser cytometer, indicating acetylcholine receptor (AChR) cluster (red), a synaptic stabilizing organization including extracellular matrix proteins (gree and light blue). The image is constructed on ACAS 570 (Meridian Instruments Inc., Okemos, MI, USA) which provides a graded pseudocolor image on the computer display. **(B)** Schematic presentation of the post-synaptic structures. Y marks attached with numbers indicate the antibodies to recognize respective targets of the functional structures. Gray frame indicates the acetylcholine receptor (AChR) cluster formation. Pink frames indicate AChR clustering by way of two signaling pathways mediated *via* the muscle-specific tyrosine kinase (MuSK) 1/2 domains (green-limit in the pink MuSK ectodomain and green line with arrowhead) and MuSK cysteine-rich domain (CRD; red-limit in the pink MuSK ectodomain and red-line with arrowhead), the signals of which are mediated by Dishevelled (Dvl, adaptor protein). The low-density lipoprotein receptor-related protein 4 (Lrp4) is the receptor for agrin (partly for Wnts as described in the text). The small GTPases (shown in the pink frame of Kinases) effector PAK1 (p21-activated kinase 1) acts as a bridging molecule between the Wnt- and agrin-signaling pathways. From “inside” the muscle cell, MuSK is activated by Dok7 (downstream kinase); Dok7 recruits two adaptor protein, Crk and Crk-L (CT10 regulators of kinase) for rapsyn-anchored AChR cluster formation. The formed AChR clusters are anchored at the endplate membrane by rapsyn and immobilized by MuSK-linking heat-shock proteins (HSPs): tumorous imaginal disc 1 short form (Tid1s), HSP 70 and HSP 90β. Tid1s is required for the MuSK-Dok7 signaling during the MuSK activation. The interaction of neuregulin 1 (NRG 1) with ErbB receptor (receptor tyrosine kinase of epidermal growth factor receptor family) increases the MuSK tyrosine phosphorylation (*via* Erbin) and thereby modulates the MuSK-dependent AChR clustering. Caveolin 3 binds with the MuSK kinase domain and thereby driving AChR clustering. Yellow frames indicate the organizations for synaptic stability and maintenance. The synaptic stability of NMJ including AChR clusters (gray frame), MuSK (pink frame), Lrp4 (pink frame) and acetylcholinesterase (AChE) is modulated by extracellular matrix proteins (collagen Q, perlecan, biglycan, laminin-network including muscle agrin and laminins and dystroglycan) worked in cooperation with the cytoskeleton. The interaction of NRG 1 (neuregulin 1) with ErbB receptor (pink frames) contributes to the cytoskeletal organization through α-dystrobrevin phosphorylation on one hand (yellow frame) and the MuSK activation *via* Erbin on the other hand (pink frame). The downstream effector of Dok7-recruited Crk-L (Sorbs1/2) acts on the cytoskeleton for synaptic stability. Collagen Q-Perlecan and Biglycan act on Dystroglycans in cooperation with cytoskeleton for synaptic stability on one hand (yellow frame) and implicate in AChR cluster formation *via* their interaction with pink-MuSK ectodomains (Ig1 shown by green limit with a green line and CRD shown by red limit with red line) on the other hand. The former is cooperated by linking to dystrophin/utrophin-associated protein complex; the latter is cooperated by linking with rapsyn to firmly anchor AChR clusters at the post-synaptic membrane. Cortactin (yellow frame) has the function of phosphorylation-dependent signaling downstream from Agrin/Lrp4/MuSK (pink frame) in promoting actin polymerization and also stabilizing AChR clusters at the postsynaptic membrane. Coronin 6 is the actin-binding protein contributive to synaptic stability. Targets recognized by antibodies: Y1, Acetylcholine receptor (AChR); Y2, Muscle-specific tyrosine kinase (MuSK) 1/2 domains; Y3, MuSK cysteine-rich domain (CRD); Y4, Low-density lipoprotein receptor-related protein 4 (Lrp4); Y5, neural Agrin; Y6, Collagen Q; Y7, Cortactin.

The neuromuscular junction (NMJ) functions as acetylcholine (ACh)-mediated synapse (Gray frame, Figure 1); its signal transmission depends on coordinated interaction between the pre-synaptic active zone (Südhof, 2012) and the post-synaptic nicotinic acetylcholine receptor (AChR; Kummer et al., 2006). In confirming the synaptic architecture, the trans-synaptic communications mediated by nerve-secreted or muscle membrane-bound proteins such as agrin (heparin sulfate proteoglycan) and Wnts (belonging to the Wingless-type integration site family of glycoproteins) play the pivotal roles *via* interactions with the muscle-specific tyrosine kinase (MuSK) and the low-density lipoprotein-related protein 4 (Lrp4; Wu et al., 2010; Shi et al., 2012; Takamori, 2013, 2017a). The review focusses on: (1) the AChR cluster formation by MuSK activation from inside (Dok-7 and Wnts) and outside (agrin and Wnts) of the muscle; (2) the synaptic compensatory mechanisms based on retrograde signals from muscle to nerve and presynaptic Ca^2+^ homeostasis by auto-receptors; and (3) the synaptic stabilization based on cytoskeletal dynamics by extracellular matrix proteins and dystrophin-associated glycoprotein complex. These have paved the way to search for the mechanisms underlying myasthenia gravis (MG) weakness (Burden et al., 2018; Sudres et al., 2018; Koneczny and Herbst, 2019). MG, an autoimmune NMJ disease characterized by fatigable weakness of voluntary muscles, are generally reviewed from the viewpoints of clinical subgroups and antibody characteristics (Vincent et al., 2018; Gilhus et al., 2019).

## Myasthenia Gravis (MG) Caused by Antibodies Targeting Key Molecules in Neuromuscular Junction

### Anti-AChR Antibodies

MG is the B cell-mediated, T cell-dependent autoimmune disease including thymic pathologies (Marx et al., 2013; Berrih-Aknin and Le Panse, 2014; Nemazee, 2017; Behin and Le Panse, 2018; Villegas et al., 2018; Yi et al., 2018; Koneczny and Herbst, 2019). In typical MG, most (~85%) patients have AChR (Gray frame in Figure 1) antibodies (Y1 in Figure 1), the pathogenic mechanisms of which are classified into (1) removal of AChRs due to cross-linking and subsequent internalization (antigenic modulation; Drachman et al., 1978), (2) functional AChR block (Drachman et al., 1982; Takamori et al., 1988) and (3) activation of complement with the formation of membrane attack complexes that cause focal lysis. The main immunogenic region targeted by MG antibodies is the AChR α-subunit 67–76 (Tzartos et al., 1982, 1988). The AChR antibodies are predominantly IgG1 and IgG3 subtypes that bind the complement causing the post-synaptic disorder in NMJ (Sahashi et al., 1980; Vincent and Newsom-Davis, 1980; Tüzün and Christadoss, 2013; Cetin and Vincent, 2018; Howard, 2018); this mechanism is most pathogenic in patients with refractory MG, who have efficaciously been treated with C5 inhibitor (eculizumab and Zilucoplan; Muppidi et al., 2019; Howard et al., 2020). Experimentally, the investigational RNAi targeting C5 (Kusner et al., 2019) and the single-chain AChR antibody coupled to complement decay-accelerating factor (Kusner et al., 2014) are shown to be efficacious in the models of MG. Promising therapeutic strategies include the B cell-related therapies (Rituximab; Tandan et al., 2017; Beecher et al., 2019; Di Stefano et al., 2020; Huda, 2020; Litchman et al., 2020) and the neonatal Fc receptor antagonists (Efgartigimod and Rozanolixizumab; Ulrichts et al., 2018; Howard et al., 2019; Huijbers et al., 2019a; Zuercher et al., 2019; Gable and Guptill, 2020). Taking into consideration of the reduced suppressive activity of T regulatory cells (Treg) in MG (reflecting on elevated inflammatory cytokines in MG; Thiruppathi et al., 2012; Ha and Richman, 2015; Wen et al., 2016; Molin et al., 2017), the Treg-based therapy achieved with the use of IL-2/anti-IL-2 monoclonal antibody complexes could be useful for treating MG as shown by the experiment in experimental autoimmune MG (Liu et al., 2010; Danikowski et al., 2017). Thymic abnormalities (hyperplasia tending to be potential in MG patients before the age of 50) have long been paid attention as an underlying pathology of MG and the target for therapy. We have participated in the MGTX (enrollment at 18–65 years of age; the disease severity of America classification class II–IV) randomized trial of thymectomy (with alternative-day prednisone), resulting in the benefit in non-thymomatous MG patients (Wolfe et al., 2019).

### Anti-striational Antigen Antibodies

The suggestive biomarkers for the presence of thymoma indicative for thymectomy are the antibodies against muscle striational antigens such as ryanodine receptor (a foot protein in the junctional gap between the dihydropyridine receptor containing T-tubules and the sarcoplasmic reticulum, functioning as Ca^2+^ release channel for E-C coupling) and titin (a protein to keep the thick filaments centered in the sarcomere, allowing optimum active force development; Mygland et al., 1992; Skeie et al., 2002; Somnier and Engel, 2002; Takamori et al., 2004; Romi et al., 2007; Romi, 2011; Choi Decroos et al., 2014; Stergiou et al., 2016). We electrophysiologically confirmed the muscular contractile (twitch and tetanus) weakness caused by anti-ryanodine receptor antibodies in the spontaneous thymoma rat (Buffalo/Mna strain suppled by Charles River Japan, Inc.; Iwasa et al., 1998).

### Immune Checkpoint Inhibitors for Cancer Therapy and MG

The therapies by use of immune checkpoint inhibitors (anti-programmed cell death protein-1 (PD1), anti-programmed death ligand-1 (PD-L1) and anti-cytotoxic T lymphocyte associated-4) to manage various cancers are occasionally associated with immune-related adverse events (due to loss of Treg homeostasis; Danikowski et al., 2017; Dalakas, 2018; Kumar et al., 2018, 2019; Möhn et al., 2019; Dubey et al., 2020) including myasthenia gravis (Becquart et al., 2019; Johansen et al., 2019; Sato et al., 2019; Choi and Lee, 2020) Reportedly, PD-L1 mRNA is highly expressed in MG muscle cells (Iwasa et al., 2018); this may suggest the participation of the local immune-mediated damage in MG muscle by the checkpoint inhibitor effect which leads to abnormal T-regulatory (Treg) cell function and altered Treg/Th17 cell axis (Dalakas, 2018). Also, PD-1 and PD-L1 are expressed in thymic epithelial tumors, suggesting a poor prognostic factor in thymomas (Bagir et al., 2018; Song et al., 2019).

### Anti-MuSK Antibodies

MuSK (Pink frame, Figure 1), a member of the receptor tyrosine kinase superfamily, is composed of four ligand-binding ectodomains (linking with neural agrin <Pink frame, Figure 1> *via* first and second immunoglobulin-like domains, Ig1/2 <green -limit in pink- MuSK, Figure 1>, and with Wnts <Blue frames, Figure 1> *via* cysteine-rich domain (CRD), <red- limit in pink-MuSK, Figure 1>, through Lrp4 <Pink frame, Figure 1>), a single transmembrane-spanning domain and an intracellular kinase domain; this receptor tyrosine kinase acts as the key organizer to form AChR clusters in the post-synaptic membrane of NMJ (Ghazanfari et al., 2011; Burden et al., 2013; Takamori, 2013; Zong and Jin, 2013; Koneczny et al., 2014; Herbst, 2019; green and red lines *via* Dishevelled (Dvl, Pink frame, Figure 1). Mesdec2 plays a key role in cell-surface expression of Lrp4 and postsynaptic specialization (Hoshi et al., 2013).

Hoch and colleagues (Hoch et al., 2001) detected the antibodies against MuSK (as discussed later, two types of antibodies are determined: Y2 targeting the Ig1/2 domains and Y3 targeting CRD in Figure 1) in the serum samples of MG patients without AChR antibodies. In general, the patients show predominant involvement in the facial, pharyngeal, tongue (often with muscle atrophy), neck, and shoulder, but extraocular muscles are rarely involved at onset in contrast to AChR antibody-positive MG; they are occasionally seized with a respiratory crisis. The patients respond to immunosuppressant therapy (Evoli et al., 2008, 2018; Pasnoor et al., 2010; Guptill et al., 2011; Evoli and Padura, 2013; Koneczny et al., 2014; Morren and Li, 2018; Li et al., 2019). The B-cell depletion therapy (Rituximab) provides more benefit to MuSK-MG patients than AChR-MG patients (Evoli et al., 2008, 2018; Díaz-Manera et al., 2012; Evoli and Padura, 2013; Tandan et al., 2017; Morren and Li, 2018; Beecher et al., 2019; Di Stefano et al., 2020; Huda, 2020; Litchman et al., 2020). In the experimental model of MuSK MG, the fetal Fc receptor antibodies (Gable and Guptill, 2020), antigen-specific immunoadsorption (Skriapa et al., 2014; Lazaridis et al., 2020) and the inhibition of tyrosine phosphatase inhibitor (SHP2; Huda et al., 2020) alleviate the MuSK antibody-induced AChR cluster deformation. One must be cautious that the hypersensitivity and worsening of symptoms to cholinesterase inhibitors tend to occur (Punga et al., 2006; Evoli and Padura, 2013). In general, thymic abnormalities are not detected and thus no apparent clinical benefit of thymectomy is obtained (Leite et al., 2005; Evoli et al., 2008; Evoli and Padura, 2013; Nakata et al., 2013; Clifford et al., 2019).

Immunologically, MuSK antibodies are mainly IgG4 subclass (Koneczny, 2018) and known to cause MG by inhibiting binding between MuSK and Lrp4 independently of complement activation (Huijbers et al., 2013), but IgG1-3 MuSK antibodies can disperse preformed agrin-independent AChR clusters (Koneczny et al., 2013).

### Anti-Lrp4 Antibodies

Lrp4 (Pink frame, Figure 1) which plays a pivotal role in mediating agrin (*via* green-limit, MuSK lg1/2, Figure 1)—and Wnt (*via* red-limit, MuSK CRD, Figure 1)—signalings for AChR clustering at the postsynaptic membrane and also serves the retrograde signaling from muscle to nerve (Barik et al., 2014a; Guarino et al., 2020). The molecular structure of Lrp4 consists of a large extracellular domain that contains eight low-density lipoprotein receptor domains class A (LDLa), EGF-like domains and four β-propeller domains; its N-terminal region (including the last few LDLa repeats and the first β-propeller domain) interacts with agrin (Zhang et al., 2011; Zong et al., 2012); in its third β-propeller domain, the edge part mediates the MuSK signaling and the central part mediates the Wnt signaling (Ohkawara et al., 2014). Autoantibodies (complement-activating IgG1 and IgG2; Y4 in Figure 1) are detected in a proportion of MG with or without antibodies to AChR and MuSK (Higuchi et al., 2011; Pevzner et al., 2011; Cossins et al., 2012; Zhang et al., 2012b; Shen et al., 2013; Zouvelou et al., 2013; Zisimopoulou et al., 2014; Marino et al., 2015; Li et al., 2017; Rivner et al., 2018; Yan et al., 2018b; Aoki et al., 2020). The severity of disease in most patients is mild to moderate, but some patients have severe symptoms causing the myasthenic crisis. Most patients respond to anti-acetylcholinesterases, steroids, and immunosuppressant medications. Thymic pathology is usually not present but underlies in some patients including thymomas (Zisimopoulou et al., 2014; Marino et al., 2015; Aoki et al., 2020). The active immunization of mice with Lrp4 was proved to induce myasthenia gravis; the pathogenicity of Lrp4 antibodies was confirmed by the study in animal models that showed both pre- and post-synaptic impairments and active complements (Mori et al., 2012, 2017; Shen et al., 2013; Ulusoy et al., 2017).

### Anti-agrin Antibodies

Agrin, a proteoglycan expressed in both motor neuron (Pink frame, Figure 1) and skeletal muscle (Yellow frame, Figure 1), is essential to form NMJ formation (Burden et al., 2018). The neural agrin-dependent pathways include the two muscle-bound transmembrane co-receptors Lrp4 and MuSK (Pink frames, Figure 1). In the functional structure of Agrin (Hoch et al., 1994; Gesemann et al., 1995; Burgess et al., 1999; Bezakova and Ruegg, 2003; Stetefeld et al., 2004; Scotton et al., 2006; Zong et al., 2012; Tezuka et al., 2014; Zhang et al., 2015), the N-terminus is required for agrin-immobilization at the NMJ; the C-terminus segment contains two of the three laminin-like G domains (G2 and G3). G2 is essential for binding of muscle agrin (Yellow frame, Figure 1) to α-dystroglycan which contributes, *via* β-dystroglycan, to the stabilization of the post-synaptic apparatus at the NMJ through the organization of the intracellular cytoskeleton of muscle (Yellow frames with black linking lines, Figure 1; muscle agrin: this is >5,000 less active compared with neural agrin as far as AChR aggregation is concerned). G3 consists of four amino acid insertion at the A/y splicing site and 8, 11 or 19 amino acid insertion at the B/z splicing site (neural agrin: Pink frame, Figure 1) and is essential for the AChR clustering activity of agrin through Lrp4-MuSK signaling (green and red lines *via* pink-framed Dvl).

Anti-agrin antibodies (Y5 in Figure 1) have been detected in some of MG patients with or without antibodies against AChR, MuSK, and Lrp4 (Gasperi et al., 2014; Zhang et al., 2014; Cordts et al., 2017; Yan et al., 2018b). Induction of anti-agrin antibodies was proved to cause MG in mice by an impairment of agrin-Lrp4 interaction (Yan et al., 2018a). We must be cautious of the antigen used for agrin-antibody assay because of the above-mentioned difference of functional structure between neural agrin and muscle agrin.

The myasthenic weakness in anti-agrin antibody-positive patients is mild to severe but tends to be moderate to the treatment, suggesting that the early detection of anti-agrin antibodies could help disease management (Cordts et al., 2017). It is worthy of note as a therapeutic device in future that a fragment of neural agrin (NT-1654) has the potential to counteract the symptoms of the diseased NMJ in the sarcopenia-like phenotype in neurotrypsin-overexpressing mice (Hettwer et al., 2014; Rudolf et al., 2014). The engineered agrin was reported to attenuate the severity of experimental autoimmune myasthenia gravis (Li Z. et al., 2018). The active form of vitamin D (1.25D) upregulates the agrin-induced AChR clustering in association with the upregulated expression of rapsyn in C2C12 myotubes (Arakawa and Wagatsuma, 2020); this pharmacologic effect could apply to a group of MG, the antibodies of which are maleficient more to rapsyn-clustered AChR than non-clustered AChR (Cetin et al., 2020).

Matrix metalloproteinases (MMPs) are membrane-anchored extracellular proteinases capable of degrading a variety of proteins including agrin; the function of agrin is regulated by the site-specific cleavage by MMPs (Patel et al., 2012; Chan et al., 2020). Additionally shown was that MMP-3 removes agrin from synaptic basal lamina (Werle and VanSaun, 2003). Reportedly, the MMP-3 level is high in the serum samples from a proportion of MG patients (Helgeland et al., 2011; Romi et al., 2012), suggesting that the NMJ might be affected by the pathogenic mechanisms as the result of the dysfunction of agrin that is brought about non-immunologically by high MMP-3 level in addition to the immune response to agrin (Luckman et al., 2011; Chan et al., 2020).

## Agrin/Wnt-Lrp4-Musk Signalings in Neuromuscular Junction

### Agrin/Dok7 Signaling *via* Lrp4/MuSK Contributes to AChR Clustering

In the ligand-binding ectodomains of MuSK, the first and second immunoglobulin-like domains (Ig1 and Ig2: Ig1/2; green limit in the pink-MuSK frame, Figure 1) mediate the agrin-Lrp4 signaling (Stiegler et al., 2006) *via* binding of the Ig1 domain (green limit in the pink-MuSK frame, Figure 1) with Lrp4 to form a tetrameric complex (Zhang et al., 2011; Zong et al., 2012). The MuSK (Pink frame, Figure 1) is stimulated from “inside” the muscle cell by an adaptor protein Dok7 (Pink frame, Figure 1) which forms a dimeric unit to dimerize and activate MuSK (Pink frame, Figure 1; Okada et al., 2006; Bergamin et al., 2010) and also recruits two adaptor proteins, CT10 regulators of the kinase (Crk and Crk-L) contributive to rapsyn (Pink frame, Figure 1)—anchored AChR cluster formation (Gray frame, Figure 1; Hallock et al., 2010). The results from the experiments to modify the Dok7 functions are such as follows: in muscles lacking Dok7, agrin failed to stimulate MuSK tyrosine phosphorylation (Inoue et al., 2009); silencing of Dok7 in rat muscle increases susceptibility to passive transfer MG (Gomez et al., 2016); overexpression of Dok7 in rat muscle enhances neuromuscular transmission with structural alterations of NMJ (Eguchi et al., 2020).

From “outside” the muscle cell, MuSK is stimulated by neural agrin (Pink frame, Figure 1) *via* Lrp4 (Pink frame, Figure 1) which is the agrin receptor and also activates MuSK without agrin (Weatherbee et al., 2006; Kim et al., 2008; Zhang et al., 2008, 2011, 2012a; Barik et al., 2014b; Stamatakou and Salinas, 2014).

### Rapsyn and Tid1s Stabilize AChR Clusters at the Membrane

The AChR clusters formed by the agrin-Lrp4-MuSK signaling are anchored at the end-plate membrane by rapsyn (Pink frame, Figure 1; Lee et al., 2009; Zuber and Unwin, 2013; Xing et al., 2020) which is immobilized by the short-form of tumorous imaginal discs-1 (Tid1s: MuSK- and Dok7-binding protein belongs to a family of heat-shock protein 40, HSP40) in cooperation with HSP70 and HSP90β (Pink frames, Figure) and thereby maintains AChR clusters (Gray frame, Figure 1) at the end-plate membrane (Linnoila et al., 2008; Luo et al., 2008; Zong and Jin, 2013). Tid1s (Pink frame, Figure 1) is also contributive to the MuSK-Dok7 signaling during the activation of MuSK (Linnoila et al., 2008). The RING domain of rapsyn contains E3 ligase activity to induce AChR clustering and NMJ formation, possibly by regulation of AChR neddylation (Li et al., 2016).

### Wnt-Mediating Signal *via* MuSK Contributes to AChR Cluster Prepatterning

In addition to the above-mentioned role as the mediator in agrin-Lrp4-MuSK signaling, MuSK acts as the mediator for Wnts (Blue frames, Figure 1) which belong to a family of secreted glycoproteins and released from motor neurons or derived from muscle (Wu et al., 2010; Gordon et al., 2012; Koles and Budnik, 2012; Zhang et al., 2012a; Barik et al., 2014b; Stamatakou and Salinas, 2014). Of 19 different Wnt molecules existing in mice and humans (Wu et al., 2010), Wnt 11 (Jing et al., 2009; Wu et al., 2010; Zhang et al., 2012a) and Wnt 3 (Henriquez et al., 2008) are released from the motor neurons; the neuron-released Wnts are transferred across the synapse by exosome-like Evi-containing vesicles (Koles et al., 2012). Wnt 11 (Wu et al., 2010; Zhang et al., 2012a), Wnt 9a (Zhang et al., 2012a), and Wnt4 (Strochlic et al., 2012; Zhang et al., 2012a) are derived from muscles. The binding of the Ig4 domain (CRD) of MuSK (red-limit in pink MuSK, Figure 1) with Wnts contributes to AChR clustering through the non-canonical pathway mediated by the Dishevelled protein (Dvl; Pink frame, Figure 1), leading to the formation of AChR microclusters at the early non-innervated stage, termed as the AChR prepatterning (Kummer et al., 2006) which forms the clusters at the central part of the muscle and guides the incoming axon (Gray frame, Figure 1; Luo et al., 2002; Jing et al., 2009; Stiegler et al., 2009; Wu et al., 2010; Gordon et al., 2012; Koles and Budnik, 2012; Barik et al., 2014b; Burden et al., 2018; Guarino et al., 2020). The motoneuron-derived Wnt7a and Wnt7b serve nerve terminal development (Shen et al., 2018).

In the clustering of AChR, Wnt 11 and Wnt 9a require Lrp4 but not agrin (Zhang et al., 2012a); Wnt 4 requires neither agrin nor Lrp4 (Strochlic et al., 2012). Wnt 3 promotes the agrin-Lrp4-MuSK (Ig1/2 domains)-mediated clustering of AChR (Henriquez et al., 2008; Wnts 11, 9a, 4 and 3: Blue frames in Figure 1). In contrast to the neuron-released Wnt 3, the muscle-derived Wnt 3a reduces the agrin-mediated AChR clustering *via* the canonical Dvl pathway to β-catenin causing the reduced rapsyn expression (Wang et al., 2008); this negative regulation maintains balance with the positive regulator (neuron-released Wnt 3) and thereby helps to sculpt the mature synaptic architecture (Henriquez and Salinas, 2012; Koles and Budnik, 2012).

Linking to the intracellular kinase domain of MuSK, the small GTPases (Rac1 and Rho) effector p21-activated kinase 1 (PAK1; Pink frame, Figure 1) acts as a bridging molecule between the Wnt- and agrin-signaling pathways required for AChR clustering (Luo et al., 2002; Wu et al., 2010; Koles and Budnik, 2012). Wnt activates Rac1 in a more efficient usage than agrin which preferentially increases Rho activity (Henriquez and Salinas, 2012).

In the innervated stage of muscle when the agrin-Lrp4-MuSK (Ig1/2 domains) signaling plays a principal role in AChR cluster formation, the cyclin-dependent kinase 5 (Cdk5) concentrated at the postsynaptic NMJ is activated and required for ACh-induced removal of prepatterning AChR clusters which are not stabilized by agrin; this is mediated through two molecular mechanisms (the upregulation of calpain activity and the interaction of Cdk5 with nestin; Lin et al., 2005; Chen et al., 2007; Mohseni et al., 2011; Yang et al., 2011; Shi et al., 2012).

### Neuregulin 1-ErbB Signaling to MuSK Activation

The ErbB receptors (receptor tyrosine kinase of EGF, epidermal growth factor, receptor family) for neuregulin 1 (NRG 1; Pink frames, Figure 1) link to the MuSK through the adaptor protein, Erbin (Simeone et al., 2010), suggesting that this signaling cascade increases the tyrosine phosphorylation of MuSK and thereby modulates MuSK-dependent AChR clustering (Ngo et al., 2012). Anti-ErbB antibodies were not detected in the sera of MG patients; however, the thymic expression of ErbB receptors was reduced in these patients (Vrolix et al., 2011).

### A Variety of Immunogenicity of MuSK

By immunoblotting against recombinant proteins (expressed in HEK 293F cells) of MuSK ectodomains, we showed that the MuSK Ig1/2 antibodies (Y2 in Figure 1) were positive in the serum samples from 33 of 43 AChR antibody-negative MG patients; 10 of them (30%) were additionally positive for MuSK CRD antibodies (Y3 in Figure 1), suggesting an impairment of both the agrin-Lrp4-MuSK (Ig1/2 domains) signaling pathway (the green line from the green limit in pink-MuSK to Kinases *via* Dvl, Figure 1) and Wnt-Lrp4-MuSK(CRD) signaling pathway (the red line from the red limit in pink-MuSK to Kinases *via* Dvl, Figure 1) in the neuromuscular transmission; however, none was positive for anti-MuSK CRD alone in our study (Takamori et al., 2013). Reportedly, the experiments by the inhibition assay in the presence of unlabelled competitive proteins (McConville et al., 2004) and the immunoprecipitation assay using MuSK Ig1 domain and CRD (Otsuka et al., 2015) suggested the co-existence of antibodies against MuSK Ig1/2 domains and MuSK CRD in MuSK antibody-positive MG. The longitudinal epitope mapping study in 53 MuSK antibody-positive MG patients by the European group showed that 22.6% of the patients were positive for MuSK CRD antibodies, although they emphasized the MuSK Ig 1 domain as the main immunogenic region (Huijbers et al., 2016). It seems likely that the MuSK antibodies have pathological heterogeneity based on their binding to functionally different domains.

To determine the functional significance of MuSK CRD in neuromuscular transmission, Messéant and associates showed that the CRD deletion of MuSK in mice caused exuberant axonal growth bypassing AChR clusters and decreased synaptic vesicle density (presynaptic impairment), and a drastic deficit in AChR clustering (postsynaptic impairment; Messéant et al., 2015). They also suggested an implication of MuSK CRD in the Wnt-canonical signaling by demonstrating that this pre- and post-synaptic impairments were rescued by lithium chloride which acts as an inhibitor of the glycogen synthese kinase-3β (Gsk3β) and an activator of the Wnt/β-catenin signaling (Messéant et al., 2015). This study was followed by the demonstration that MuSK ΔCRD is malfunctioning since MuSK^ΔCRD/−^ embryos present defective pre- and post-synaptic differentiation (Boëx et al., 2018). In addition to the above-mentioned Wnt-canonical signaling pathway, they proposed the β catenin-dependent and vangl 2 (coimmunoprecipitated with Wnts 4 and 11 in muscle)-dependent Wnt non-canonical signaling pathway (Messéant et al., 2017). The experiment in mice suggested that the NMJ pre- and post-synaptic organizations by β-catenin-dependent or Wnt-dependent signaling is regulated by Yes-associated protein (Yap) in the muscle (Zhao et al., 2017). Reportedly, the inhibition of the MuSK-Dvl signaling pathway (Wu et al., 2010; Koles and Budnik, 2012) decreased not only the amplitude of spontaneous synaptic currents but also their frequency (Luo et al., 2002), suggesting a defect in ACh-release upregulation to compensate for the post-synaptic dysfunction (Takamori et al., 1984; Plomp et al., 1995; Plomp, 2017; Takamori, 2019). This, in turn, may reflect an impairment in the retrograde Wnt-MuSK (CRD)-Dvl-Gsk3β inhibition-β catenin-slit 2 signaling at the nerve terminal (Luo et al., 2002; Salinas, 2005; Li et al., 2008; Budnik and Salinas, 2011; Koles and Budnik, 2012; Wu et al., 2012a, 2015; Barik et al., 2014b). Postsynaptically shown in the experiment is that β catenin regulates agrin-induced AChR clustering through interaction with rapsyn in a manner dependent on α catenin-associated cytoskeleton (Zhang et al., 2007). These can help explain the reports showing both pre- and post-synaptic defects in MuSK antibody-positive MG patients (Niks et al., 2010) and animal models (Cole et al., 2008; Klooster et al., 2012; Mori et al., 2012; Richman et al., 2012; Viegas et al., 2012). In the study of congenital myasthenic syndrome due to MuSK mutation, the changes in the presynaptic morphology were assumed to reflect a disturbance in muscle-derived retrograde signals resulting from the impairment of agrin- and Dok7-induced MuSK phosphorylation (Maselli et al., 2010; Ben Ammar et al., 2013).

We noted the debate by Remédio and associates who proposed that dependently on species, the prepatterning of AChR clusters is influenced by an additional presence of Kringle domain in the MuSK extracellular region (Remédio et al., 2016; Burden et al., 2018); this is likely to augue against the participation of MuSK CRD in signaling variety in MuSK antibody-positive MG patients. In this regard, Legay and Mei (2017) suggested that the controversial result may reflect the genotype-phenotype relationships because opposite phenotypes can be found depending on the genetic background (Sittig et al., 2016).

In the MuSK antibody-positive MG patients, the recent research reported that the monovalent MuSK antibodies abolished agrin-induced MuSK phosphorylation and AChR clustering, while the divalent monospecific MuSK antibodies had the opposing effect which leads to partially induced AChR clustering; this is independent of agrin (Huijbers et al., 2019b). It is also noticeable that Takata and his associates generated human MuSK monoclonal autoantibodies (2 IgG4 and 1 IgG3 subclasses obtained using isolation of MuSK autoantibody-expressing B cells from MuSK antibody-positive MG patients) and showed that these antibodies inhibited AChR clustering, but enhanced MuSK phosphorylation; they suggested an alternative mechanism for inhibiting AChR clustering (Takata et al., 2019).

### Bone-Morphogenic Proteins (BMPs) and MuSK Activation

The BMPs, which are secreted ligands belonging to the transforming growth factor β (TGFβ) superfamily of signaling proteins, have been focused in highlighting insight into the possibility that BMPs work in vertebrate organisms as diffusible signals to ensure proper communication between motor neurons and skeletal muscles (Osses and Henriquez, 2015). In a variety of function and localization of BMPs, it is an interest to note the reports that the Wnt-MuSK Ig3 domain interaction acts as a BMP co-receptor which shapes BMP transcriptional output and cholinergic signaling to communicate motor neurons and skeletal muscles in the vertebrate (Henríquez et al., 2011; Yilmaz et al., 2016). The BMP-4 has been confirmed to be expressed in the skeletal muscle and localized in close vicinity to postsynaptic densities at the NMJ (Chou et al., 2013). Further work may provide a clue to the understanding of BMPs-mediated pathology in MuSK antibody-positive MG patients.

## Synaptic Retrograde Signalings from Muscle to Nerve

### Wnt-MuSK, Lrp4, and Laminins

As previously discussed in a variety of immunogenicity of MuSK, the interaction of Wnt with MuSK CRD leading to Dvl-GSK3β inhibition-β catenin-Slit 2 signaling (Luo et al., 2002; Salinas, 2005; Li et al., 2008; Budnik and Salinas, 2011; Koles and Budnik, 2012; Wu et al., 2012a; Barik et al., 2014b; Messéant et al., 2015) and the muscle Lrp4 (extracellular eight low-density lipoproteins repeats; Wu et al., 2012b; Yumoto et al., 2012) contribute to the retrograde signaling and thereby modulate the pre-synaptic differentiation including synaptic vesicles and active zone in the nerve terminal. Additional retrograde signaling in the synapse is mediated by the muscle-derived laminins α4, α5, and β2 which contribute to the presynaptic organization acting for ACh release; this includes the action of laminin β2 which tethers P/Q- and N-types voltage-gated Ca^2+^ channels (VGCCs) to the active zone proteins (Nishimune et al., 2008; Carson et al., 2010; Chen et al., 2011; Nishimune, 2012; Samuel et al., 2012; Yamada and Sekiguchi, 2015; Zhang et al., 2015; Rogers and Nishimune, 2017).

### Presynaptic Ca^2+^ Homeostasis and Autoreceptors

From the standpoint of clinical electrophysiology, the AChR antibody-positive MG and animal models showed that the compensatory ACh release upregulation cannot be sustained at the high frequency of nerve stimulation (Plomp et al., 2015). This may reflect the impairment of the presynaptic Ca^2+^ homeostasis for ACh release (Takamori, 2012) to compensate for postsynaptic dysfunction. The presynaptic Ca^2+^ homeostasis is promoted by the activation of G protein-coupled receptors such as M1-type muscarinic AChR (M1 mAChR) and A2A adenosine receptor along with the interaction of brain-derived neurotrophic factor (BDNF) with receptor tyrosine kinase B (TrkB); these biological mechanisms lead to P/Q-type VGCC activation by the signaling mediated by phospholipase C (PLC)-phosphatidylinositol 4,5-bisphosphate (PIP2)-diacylglycerol (DAG)-protein kinase C (PKC; Amaral and Pozzo-Miller, 2012; Santafé et al., 2015; Nadal et al., 2016; Hurtado et al., 2017; Simö et al., 2018; Tomàs et al., 2018). Also, PLC-generated DAG regulates the presynaptic vesicle priming protein (Munc13-1) to recruit ACh-containing vesicles for the immediately releasable pool (Bauer et al., 2007). Taking these into consideration, the presynaptic impairments to compensate postsynaptic dysfunction could, at least in part, be compatible with the result of our immunological study that the M1 mAChR antibodies were positive in 7 (28%) of our 25 post-synaptic nicotinic AChR antibody-positive MG patients (Takamori et al., 2007; Takamori, 2019); additionally, the anti-M1 mAChR antibodies were often (76%) positive in our 25 patients with Lambert-Eaton myasthenic syndrome (an autoimmune presynaptic disease caused by antibodies against mainly P/Q-type VGCC and partly synaptotagmin 1; Takamori et al., 2007; Takamori, 2008a). Of note, the M1 mAChR is physiologically associated with the non-voltage-gated Ca^2+^-dominant influx channel (Transient receptor potential canonical; Kim and Saffen, 2005), the antibodies against which were positive in 7 (28%) of our 25 MG patients (Takamori, 2008b). An alternative theory postulated is that the impaired compensatory mechanism could be referred to the reduction in homeostatic small reserve pool of synaptic vesicles that depends on presynaptic adenosine A2A receptor operating Ca^2+^ influx *via* L-type VGCC (Oliverira et al., 2004; Ma et al., 2015; Wang et al., 2016). In a long-tetanic load in muscle, the complex compensatory mechanisms including the presynaptic autoreceptors and Ca^2+^ influx channel(s) may underlie to compensatory postsynaptic dysfunction.

## Therapeutic Option of β_2_-Adrenoceptor Agonists in Myasthenias

Besides the biological signal (compensatory presynaptic Ca^2+^ homeostasis) triggered by G protein-coupled receptor, it is worthy to note that BDNF in the synapse is activated by β_2_-adrenoceptor agonists and thereby contributes to the maintenance of the structural and functional integrity of motor end-plate; this signaling pathway may include the cyclic adenosine monophosphate (cAMP)/protein kinase A/cAMP-responsive element-binding protein; Bartus et al., 2015). The therapeutic pre- and post-synaptic benefits of β_2_-adrenoceptor agonists (salbutamol and ephedrine) have been reported in MuSK MG animal model (Ghazanfari et al., 2014), autoimmune MG patients (Rodríquez Cruz et al., 2015b; Lipka et al., 2017; Vanhaesebrouck et al., 2019) and various types of congenital myasthenic syndromes (Engel et al., 2015; Beeson, 2016) including the patients with mutations in MuSK (Gallenmller et al., 2014; Rodríquez Cruz et al., 2020; Vanhaesebrouck and Beeson, 2019), Lrp4 (Selcen et al., 2015; Beeson, 2016), Dok7 (Lashley et al., 2010; Liewluck et al., 2011; Burke et al., 2013; Lorenzoni et al., 2013), agrin (Nicole et al., 2014; Beeson, 2016; Vanhaesebrouck and Beeson, 2019), collagen Q (McMacken et al., 2019) and collagen XIII (Beeson, 2016; Rodríguez Cruz et al., 2018, 2019; Dusl et al., 2019). In pharmacotherapeutic options, the β_2_-agonist stimulation could be chosen as a novel therapeutic strategy in autoimmune and genetic myasthenias.

## Candidates as The Molecules Implicating in Pathogenicities of Myasthenias

The studies including clinical researches may suggest the following molecules as candidates to implicate in the pathogenic mechanisms of myasthenic diseases: (1) caveolin 3 (Pink frame, Figure 1; MuSK kinase domain-binding protein which participates in agrin-induced phosphorylation and activation of MuSK, thereby driving AChR clustering; Hezel et al., 2010; Iwasa et al., 2016); (2) doublecortin (expressed in motor neurons and skeletal muscles to orderly form the pre- and post-synaptic morphology; Bourgeois et al., 2015); (3) R-spondin 2 (expressed in motor neurons and reactive with leucine-rich repeat-containing G-protein-coupled receptor which is expressed in skeletal muscles and enriched in the NMJ so as to regulate Wnt-mediated AChR clustering and also acting on an effect for synaptic vesicle recycling and number of active zones in the nerve terminal; Vieira et al., 2015; Nakashima et al., 2016); (4) amyloid precursor protein (APP)/APP-like protein (contributive to AChR clustering *via* the Lrp4-MuSK signal in cooperation with agrin-mediated signal and also to the presynaptic differentiation of the NMJ, and additionally contributive to the density of ACh-containing synaptic vesicles which is modulated by glia cell-derived neurotrophic factor, GDNF, expressed in muscle cells; Akaaboune et al., 2000; Wang et al., 2009; Caldwell et al., 2013; Choi et al., 2013; Stanga et al., 2016), and (5) α-neurexins (contributive to neurotransmission and differentiation of the synapse by functioning as the trans-synaptic complex with postsynaptic proteins such as neuroligins (Sons et al., 2006; Reissner et al., 2013; Plomp, 2017).

## Synaptic Stability and Maintenance by Extracellular Matrix and Dystrophin-Associated Glycoprotein Complex

To be precisely opposed to the nerve terminal including the active zone artitecture (Nishimune, 2012; Südhof, 2012; Harris and Littleton, 2015; Körber and Kuner, 2016), the postsynaptic structure is stabilized by the extracellular matrix proteins (including synaptic collagens such as Q, IV and XIII, perlecan, biglycan, laminins, muscle agrin, nidogens and α-dystroglycan; Yellow frames, Figure 1), dystrophin-associated glycoprotein complex (including β-dystroglycan which links to rapsyn and utrophin; Yellow frames, Figure 1), cortactin (Yellow frame, Figure 1; cortactin-directing antibodies: Y7 in Figure 1; acting as not only a synaptic kinase substrate but also a regulator of actin polymerization), coronin 6 (Yellow frame, Figure 1; actin-binding protein which plays a role in synaptic stability), α-dystrobrevin (Yellow frame, Figure 1; contributive to cytoskeletal organization due to its phosphorylation by neuregulin 1(NRG 1)-ErbB receptor interaction: Pink frames in Figure 1) and Sorbs 1/2 (Yellow frame, Figure 1; contributive to synaptic stabilization by its interaction with Dok7-recruited Crk-L Nishimune et al., 2008; Madhavan et al., 2009; Hallock et al., 2010, 2015; Pilgram et al., 2010; Schmidt et al., 2011; Singhal and Martin, 2011; Yurchenco, 2011; Amenta et al., 2012; Maselli et al., 2012; Nastase et al., 2012; Shi et al., 2012; Hochenester and Yurchenco, 2013; Karmouch et al., 2013; Chen et al., 2014; Constantin, 2014; Takamori, 2017a,b; Belhasan and Akaaboune, 2020). The following is the synaptic partners organizing molecules that regulate synaptic stability.

### Collagen Q (Col Q) and Perlecan

Collagen Q (Yellow frame, Figure 1) is one of the muscle basal lamina proteins (junctional collagens; Singhal and Martin, 2011). Its N-terminus interacts with acetylcholinesterase (AChE); the two heparin-binding sites of its central collagenic domain mediate the interaction with perlecan (Yellow frame, Figure 1) which binds α-dystroglycan (Yellow frame, Figure 1) for linking the extracellular matrix to the cytoskeleton (Yellow frame, Figure 1). Its C-terminus binds the Ig1 domain (green limit) and CRD (red limit) in MuSK (Pink frame, Figure 1); these links make ColQ (*via* green and red lines, Figure 1) to modulate the action of the other ligands of the MuSK/Lrp4 complex for NMJ formation (Karmouch et al., 2013). The ColQ anchors AChE in the synaptic basal lamina, and thereby the association between Col Q and MuSK conforms Col Q/AChE to the synaptic localization (Cartaud et al., 2004). The MuSK antibodies were found to block the Col Q-MuSK interaction (Kawakami et al., 2011). Reportedly, the Col Q and MuSK antibodies competitively suppress agrin/Lrp4/MuSK signaling (Otsuka et al., 2015). Col Q antibodies (Y6 in Figure 1) were detected in some MG patients positive for MuSK and AChR antibodies (Cossins et al., 2012; Zoltowska Katarzyna et al., 2015). These findings are compatible with the electrophysiological finding characterized by slow miniature end-plate potential kinetics and hypersensitivity to AChE inhibitors in MuSK antibody-positive MG (Shin et al., 2014) and its animal model (Mori et al., 2012).

### Collagen XIII

Collagen XIII is encoded as an extracellular matrix protein and is known to be crucial for the formation and function of pre- and post-synaptic organization of the NMJ, including the synaptic integrity through binding to the Col Q tail of AChE, AChR clustering, synaptic vesicle accumulation and axonal neurofilament (Latvanlehto et al., 2010; Logan et al., 2015; Härönen et al., 2017, 2019; Beeson et al., 2018; Heikkinen et al., 2019; Rodríquez Cruz et al., 2019). Autoantibodies to the collagen XIII have recently been reported in MG (Tu et al., 2018), suggesting the test as to if the Collagen XIII could be a pathogenic antigen in MG patients.

### Biglycan

Biglycan (Yellow frame, Figure 1) is one of the proteoglycans and is enriched in the postsynaptic membrane and is a ubiquitous structural component of the extracellular matrix, and also acts as signaling molecules (Amenta et al., 2012; Nastase et al., 2012). The glycosaminoglycan-binding form of biglycan mediates its binding to extracellular α-dystroglycan (Yellow frame, Figure 1); the transmembrane β-dystroglycan (Yellow frame, Figure 1) binds to rapsyn (Pink frame, Figure 1) for firmly anchoring AChR clusters at the postsynaptic membrane (from Yellow frame to Pink frame, being connected by a black line, Figure 1) and also binding to the dystrophin/utrophin (Yellow frame, Figure 1)—associated protein complex contributive to the organization of cytoskeleton (Yellow frame, Figure 1) for synaptic stability. The non-glycosylated form of biglycan (lacking glycosaminoglycan side chain) directly interacts with two extracellular domains of MuSK: one (green line) is the agrin/Lrp4-mediating Ig1 domain of MuSK (green limit in pink-MuSK, Figure 1; Weatherbee et al., 2006; Kim et al., 2008; Zhang et al., 2008, 2011; Zong et al., 2012; Zong and Jin, 2013) and the other (red line) is the Wnt-mediating MuSK CRD (red limit in pink-MuSK, Figure 1; Jing et al., 2009; Koles and Budnik, 2012; Strochlic et al., 2012; Zhang et al., 2012a). The evidence that the two sites of MuSK ectodomain bind biglycan suggest possible participation of this matrix protein in reinforcing a functional bridge between the agrin-signaling (*via* Ig1 domain) and Wnt-signaling (*via* CRD; Barik et al., 2014b). Since biglycan becomes incapable of linking to MuSK by mutations of Ig1 domain and CRD (Amenta et al., 2012), the interaction between MuSK and biglycan (non-glycanated form) is potentially blocked by MuSK antibodies in the manner similar to the block of the MuSK-Col Q linkage by MuSK antibodies (Kawakami et al., 2011). In the serum samples from MG patients that we previously studied for the detection of antibodies recognizing MuSK Ig1/2 domains and CRD (Takamori et al., 2013), we tested if they harbor antibodies that directly recognize biglycan, but obtained the negative result (Takamori, 2017b). However, the MuSK antibodies may impair the synaptic stability by the disturbed MuSK-biglycan linkage. In addition to the immunological participation, biglycan stimulates multifunctional proinflammatory signaling linking the innate to adaptive immune systems when it is in a soluble form; this is based on that biglycan is capable of clustering several types of pathogen recognition receptors and orchestrating their signalings (Bavelova et al., 2009; Moreth et al., 2012; Nastase et al., 2012; Frey et al., 2013). In MG, biglycan produced in thymic myoid cells is known to play in generation and maintenance of the hyperplastic change of MG thymus (Tomoyasu et al., 1998; Takamori, 2017b).

### Cortactin

Cortactin (Yellow frame, Figure 1) is a protein that is a tyrosine kinase substrate and regulates actin polymerization, and is co-enriched at AChR clusters; its tyrosine phosphorylation is enhanced by agrin, suggesting a novel function of phosphorylation-dependent cortactin signaling downstream from agrin/MuSK in promoting actin polymerization (through actin-related proteins 2/3 complex activation) and stabilizing AChR cluster at the postsynaptic membrane (Madhavan et al., 2009). Cortactin antibodies (Y7 in Figure 1) were detected in some of the MG patients (Gallardo et al., 2014; Cortés-Vicente et al., 2016; Illa et al., 2018), and also found in a patient with Lambert-Eaton myasthenic syndrome (a presynaptic autoimmune disease; Gallardo et al., 2014); this may reflect the fact that cortactin expresses in the presynaptic side (Peng et al., 1997) and acts as the Wnt signaling-dependent presynaptic effector molecule (Alicea et al., 2017).

## Conclusion and Perspective

We emphasize: (1) the AChR cluster formation based on the Wnts-Lrp4-MuSK (CRD)-Dvl signaling (pre-innervated stage) which converges upon the agrin-Lrp4-MuSK (Ig1/2 domains) pathway (innervated stage); (2) the retrograde signaling (from muscle to the nerve) for presynaptic differentiation *via* MuSK CRD, Lrp4 and Laminins; (3) presynaptic Ca^2+^ homeostasis by autoreceptors; and (4) the synaptic stability based on the laminin-network including the extracellular matrix and the cytoskeleton. The insight into the NMJ structural mechanisms discussed in this review will foster further immunological approaches to search for new antigenic targets (Li L. et al., 2018; Koneczny and Herbst, 2019) and to enhance antibody detection (Vincent et al., 2012, 2018; Rodríguez Cruz et al., 2015a; Huda et al., 2017; Lazaridis and Tzartos, 2020) in MG, and may thereby help to view clinical profiles in MG subtypes (Verschuuren et al., 2013; Gilhus et al., 2019) and to provide potential therapeutic approaches (Losen et al., 2005; Ghazanfari et al., 2014; Gomez et al., 2014; Dalakas, 2015, 2019; Behin and Le Panse, 2018; Ito and Ohno, 2018; Morren and Li, 2020).

## Author Contributions

The author confirms being the sole contributor of this work and has approved it for publication.

## Conflict of Interest

The author declares that the research was conducted in the absence of any commercial or financial relationships that could be construed as a potential conflict of interest.
